# Resveratrol-loaded metal-organic framework for mitochondria-targeted amplified CO gas therapy

**DOI:** 10.3389/fchem.2025.1545850

**Published:** 2025-01-20

**Authors:** Fengqin Wang, Yinfang Jiang, Yang Wang

**Affiliations:** School of Mechanical Engineering, Nantong Institute of Technology, Nantong, Jiangsu, China

**Keywords:** metal-organic framework, mitochondria-targeting, reactive oxygen Species, combined therapy, gas therapy

## Abstract

Carbon monoxide (CO) based gas therapy has recently garnered significant attention due to its remarkable therapeutic effects for various major diseases. However, the primary challenge in gas therapy is the effective delivery of gas prodrug to targeted sites, as well as achieving precise spatial-temporal control over their release behavior. In this work, we provide a facile method to design ROS-responsive and mitochondrial targeting CO-delivery nanoplatform, based on the thiol-functionalized metal-organic framework (MOF), abbreviated as UiO-66-SH, incorporating the drug resveratrol (RES) for combined tumor therapy. After endocytosis by tumor cells and localization within the mitochondria, UiO@FeCO@RES was decomposed by ATP to release RES and generate CO gas via a Fenton-like reaction between hydroxyl radicals (·OH) and FeCO. RES acts as an ATPase inhibitor, disrupting the metabolism of the respiratory chain in tumor cell and thereby facilitating ATP-blocked metabolic therapy. *In vitro* experimental results demonstrate that the combination therapy, involving both RES drug and CO gas therapy, exhibits a synergistic effect against cancer cells. This synergistic strategy has endowed UiO@FeCO@RES as a promising material for biomedical applications.

## 1 Introduction

Gas therapy, an emerging treatment paradigm that utilizes nearly nontoxic gasotransmitters to induce cancer cell death, has garnered significant attention (He, 2017; Yu et al., 2018; Chen et al., 2019; Wang et al., 2020; Jin et al., 2021; Yao et al., 2022). This approach is regarded as a “green” treatment option, characterized by minimal adverse toxicity to normal organs, while providing substantial metabolic benefits that are often unattainable through chemotherapy or other conventional therapeutic modalities (Manoharan et al., 2019; Zhang et al., 2020; Jing et al., 2021). Various gaseous molecules, including hydrogen (H_2_), nitric oxide (NO), carbon monoxide (CO), and hydrogen sulfide (H_2_S), have been recently utilized as therapeutic agents in cancer treatment (Yu et al., 2018; Huang et al., 2021; Wang et al., 2021). Remarkably, CO acts as an endogenous gasotransmitter produced through heme-oxidation and is vital in various physiological functions (García-Gallego and Bernardes, 2014). It has a direct adverse effect on mitochondrial function by disrupting the activity of relevant enzymes to block the production of adenosine triphosphate (ATP), thereby achieving the inhibition of cell growth and proliferation (Motterlini and Otterbein, 2010; Wu et al., 2020; Zhou et al., 2020; Zhang et al., 2022; Wang et al., 2024). CO gas can also synergistically improve the effects of traditional treatments, including chemotherapy, radiotherapy, photothermal therapy (PTT), and photodynamic therapy (PDT) (Tang et al., 2023; Wang et al., 2023b; Zhang et al., 2023; Zhou et al., 2023; Tang et al., 2024; Zhou et al., 2024). For instance, CO gas may help to address the issue of drug resistance commonly encountered in traditional chemotherapy by inhibiting ATP production and downregulating the expression of drug transporter proteins (Yan et al., 2019; Zhou et al., 2020). Therefore, the development of ideal therapeutic platforms with safe, accurate, and controllable delivery of CO to tumor sites still remains to be a desirable goal.

Mitochondria, as essential subcellular organelles, are responsible for producing ATP, controlling intracellular redox homeostasis, and regulating cytosolic calcium levels (Liew et al., 2021; Qian et al., 2024). Moreover, mitochondria initiate cell apoptosis by activating the formation of mitochondrial permeability transition pore and are involved in cell proliferation and differentiation (Guo et al., 2021; Xu et al., 2022). Given their vital roles, mitochondria have emerged as significant targets for cancer treatment strategies. Certain pharmacological agents, such as resveratrol (RES), an ATPase inhibitor, can directly affect mitochondrial function by suppressing the aerobic respiratory chain in tumor cells, consequently hindering ATP production for metabolic therapy (Wan et al., 2020; Wang et al., 2023a; Zhang et al., 2023; Tang et al., 2024). Therefore, the design of a mitochondria-targeting therapeutic nanoplatform by the combination of CO gas and RES might be a promising strategy for cancer therapy.

**SCHEME 1:**
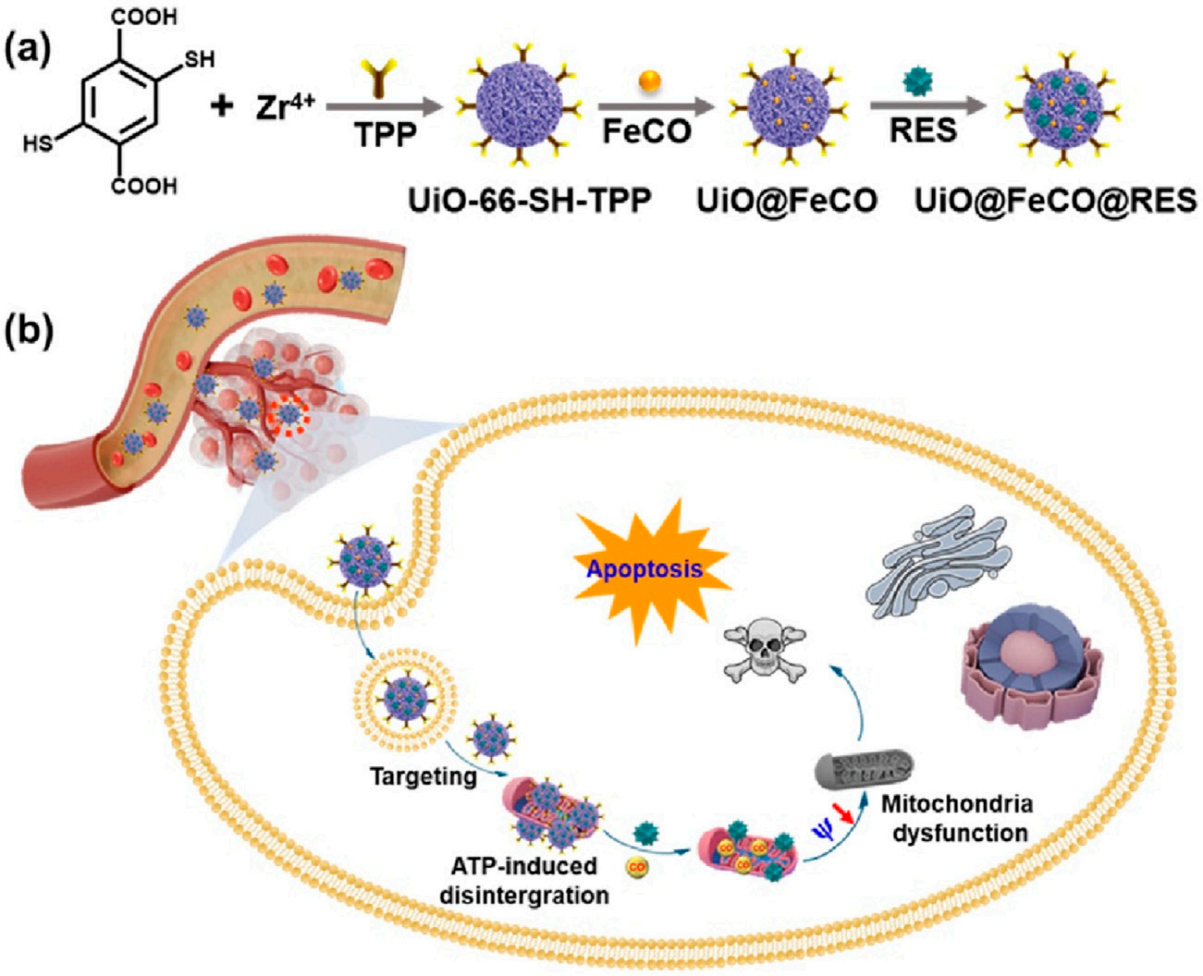
Schematic illustration of **(A)** the synthesis of UiO@FeCO@RES, and **(B)** application for enhanced CO gas therapy of cancer cells.

Herein, we propose a targeted delivery and controlled release strategy, designing an intelligent nanotherapeutic agent, UiO@FeCO@RES, to achieve the mitochondria-targeted delivery of the nanomedicine, the ATP-triggering decomposition, and the intramitochondrial reactive oxygen species (ROS)-responsive release of CO gas (Scheme 1A). As illustrated in Scheme 1B, following endocytosis by cancer cells, the UiO@FeCO@RES nanoplatform can effectively localize to the mitochondria. It is subsequently decomposed by ATP overexpressed in the mitochondria, leading to the release of RES for ATP-blocked metabolic therapy through suppression of the aerobic respiratory chain. Concurrently, a Fenton-like reaction occurs between ·OH and CO-prodrug of iron carbonyl (FeCO) to generate CO gas in mitochondria, which induces mitochondrial dysfunction and ultimately results in cell apoptosis. This combination therapy by synergistic effects of RES and gas therapy presents the superiority of the treatment strategy and paves the way for novel cancer treatment models in the future.

## 2 Experiment section

### 2.1 Synthesis of UiO-66-SH-TPP, UiO@FeCO, and UiO@FeCO@RES

#### 2.1.1 UiO-66-SH-TPP

2,5-Dimercapto-1,4-benzenedicarboxylic acid (H_2_DMBD) was synthesized following previously established protocols (Yee et al., 2013). ZrCl_4_ (41.2 mmol) was dissolved in *N,N*-dimethylformamide (DMF, 10 mL) containing acetic acid (750 μL) under ultrasonic agitation for 30 min to ensure complete dissolution. H_2_DMBD (41.3 mmol) and (4-carboxybutyl) triphenylphosphonium bromide (TPP, 11.5 mmol) were subsequently added to the solution, and the mixture was sonicated for an additional 30 min. The resulting solution was then transferred to a Teflon-sealed autoclave and heated to 120°C for 24 h. After natural cooling, the sample was centrifuged and washed with anhydrous methanol for several times to remove the residual DMF. Then, the TPP-functional MOF (UiO-66-SH-TPP) was obtained. The preparation method for UiO-66-SH was identical to that described above, with the exception of the inclusion of TPP.

#### 2.1.2 UiO@FeCO

A total of 10 mg of UiO-66-SH-TPP was added to a 50 mL tetrahydrofuran (THF) solution containing 20 mg of Fe_3_(CO)_12_ (FeCO), and the mixture was sonicated for 15 min. Subsequently, the mixture was stirred and heated to 70°C for 1 h. Upon completion of the reaction, the product was collected via centrifugation and carefully rinsed with anhydrous methanol to remove any residual FeCO. The final product was then vacuum-dried and stored in the dark.

#### 2.1.3 UiO@FeCO@RES

5 mg of UiO@FeCO was dispersed in a 10 mL methanol solution containing 5 mg of resveratrol (RES). The mixture was stirred gently for 12 h under dark. Subsequently, the products were collected by centrifugation and washed three times with methanol. Finally, the resulting UiO@FeCO@RES nanoparticles were vacuum-dried and stored in the dark.

### 2.2 ROS-responsive CO gas release from UiO@FeCO@RES

The release of CO gas in PBS was detected spectrophotometrically by monitoring the conversion of hemoglobin (Hb) to carboxyhemoglobin (HbCO) (Meng et al., 2020; Wang et al., 2024). Initially, Hb was completely dissolved in PBS (pH 7.4) and subsequently reduced by the addition of sodium dithionite under a nitrogen atmosphere. Following this, UiO@FeCO@RES and a fresh mixture of ferrous sulfate (FeSO_4_) and hydrogen peroxide (H_2_O_2_) were sequentially added into the Hb solution, to generate ·OH via the Fenton reaction, thereby triggering CO release from the prodrug. Various amounts of hydroxyl radicals were produced by adjusting the amounts of FeSO_4_ and H_2_O_2_ used. The UV adsorption spectrum of the solution was measured using a Cary 60 UV/Vis spectrophotometer.

### 2.3 Release of RES in response to ATP

A concentration of 2.0 mg mL^−1^ UiO@FeCO@RES nanoparticles was suspended in PBS at pH 7.4, both in the presence or absence of 1.0 mg mL^−1^ ATP. The mixture was shaken at 37°C with a speed of 200 rpm for varying durations. Subsequently, 2 mL of buffer solution was removed and replaced with an equal volume of fresh buffer. Finally, the absorbance of RES at 325 nm in buffer was measured using UV–vis absorption spectrometer. The cumulative amount of RES released from RES@PCN was determined based on the standard curve of RES.

### 2.4 Colocalization study of UiO@FeCO@RES with mitochondria

HeLa cells were seeded in small dishes for 24 h incubation. Rh 6G-labeled nanoparticles (UiO-66-SH and UiO-66-SH-TPP) were then added and incubated with the cells for 4 h. Soon after, the cells were washed with PBS and stained with Mito Tracker Red. After 45 min, the cells were washed again and observed using CLSM.

### 2.5 Measurement of mitochondrial membrane potential

The mitochondrial fluorescent probe JC-1 was employed to investigate the change of the mitochondrial membrane potential (MMP). HeLa cells were seeded in 6-well plates and incubated for 24 h. The medium was replaced with fresh medium containing UiO-66-SH-TPP, UiO-66-SH@FeCO@RES, and UiO@FeCO@RES. After a 6-hour incubation period, a JC-1 solution (1.0 μg mL^−1^) was added and incubated with cells for 20 min. Subsequently, the cells were washed three times with PBS and observation using a fluorescent microscope.

### 2.6 MTT assays

All cell procedures were conducted with the approval of the Nantong Institute of Technology of ethics committee. The cytotoxicity of UiO-66-SH-TPP was assessed using the MTT assay with HeLa cells. In brief, HeLa cells were seeded into 96-well plates at a density of 1 × 10^5^ cells per well and incubated for 24 h. Subsequently, the cells were treated with various concentrations of RES, UiO@FeCO, and UiO@FeCO@RES ranging from 10 to 160 μg mL^−1^, in DMEM for an additional 24 h. Following this incubation, the culture medium was replaced with 200 μL of DMEM containing a 5 mg mL^−1^ MTT solution (10 μL). After 4 h incubation period, 150 μL of DMSO was added, and the mixture was agitated for 10 min to dissolve blue formazan crystals. The absorbance of each well at 490 nm was subsequently measured using a microplate reader.

### 2.7 Statistical analysis

All data were provided as mean ± standard deviation (SD). The graphs were made using Origin 2021. The statistical significance of different groups was given by using Student’s t-test.

## 3 Results and discussion

### 3.1 Synthesis and characterization of UiO@FeCO@RES

**FIGURE 1:**
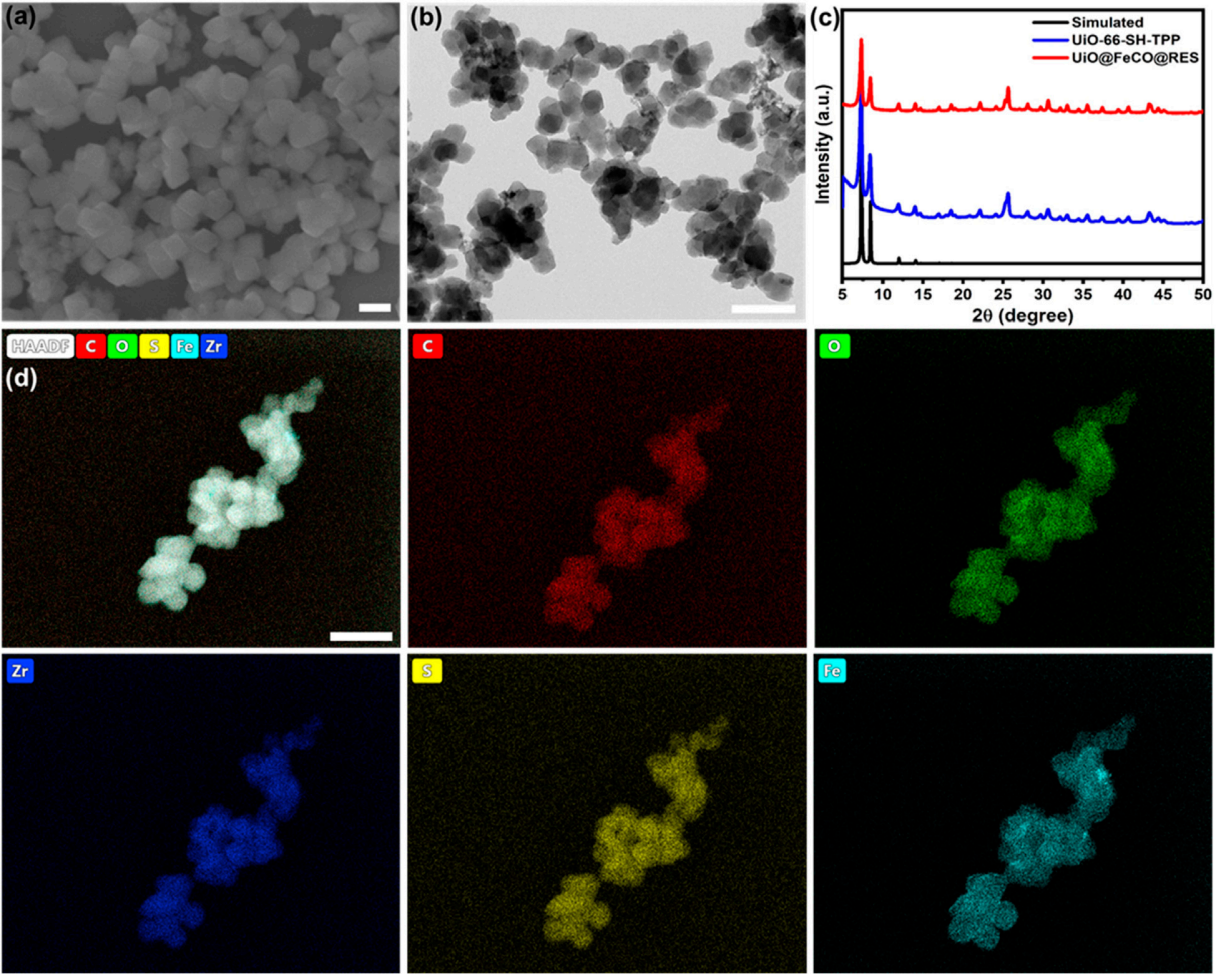
UiO@FeCO@RES NPs: **(A)** SEM image, **(B)** TEM image, **(C)** PXRD pattern, **(D)** Element mapping. Scale bar = 200 nm.

As illustrated in Scheme 1A, TPP-functionalized MOFs (UiO-66-SH-TPP) were synthesized using a one-step solvothermal approach, following previously established protocols (Yee et al., 2013). The CO prodrug of FeCO was subsequently integrated into the thiol-functionalized coordination network of UiO-66-SH-TPP through a coordination linkage reaction, resulting in the formation of UiO@FeCO. Additionally, due to its porous structure, the drug RES was successfully loaded into the pores, yielding the nanotherapeutic agent of UiO@FeCO@RES. As illustrated in Figures 1A, B, the SEM and TEM images revealed that the designed nanotherapeutic agent of UiO@FeCO@RES exhibited a relatively uniform dispersion and a regular octahedral morphology, with a mean diameter of approximately 120 nm. Figure 1C presents a comparison of the PXRD patterns measured for UiO-66-SH-TPP and UiO@FeCO@RES against the simulated PXRD pattern for UiO-66. Identical characteristic XRD peaks were observed for both samples, indicating that they share the same topology as UiO-66 (Cavka et al., 2008). Furthermore, the results demonstrated that the original structure of the MOFs remained intact after the incorporation of FeCO. The composition of the UiO@FeCO@RES nanoparticles was confirmed using EDX element mapping, as shown in Figure 1D, which indicated that the Fe elements were uniformly distributed throughout the MOF structure, thereby confirming the successful loading of FeCO. Importantly, the size distribution of UiO@FeCO@RES did not alter significantly after storage in PBS solution for 3 days, suggesting their good dispersity and stability (Supplementary Figure S1).

**FIGURE 2:**
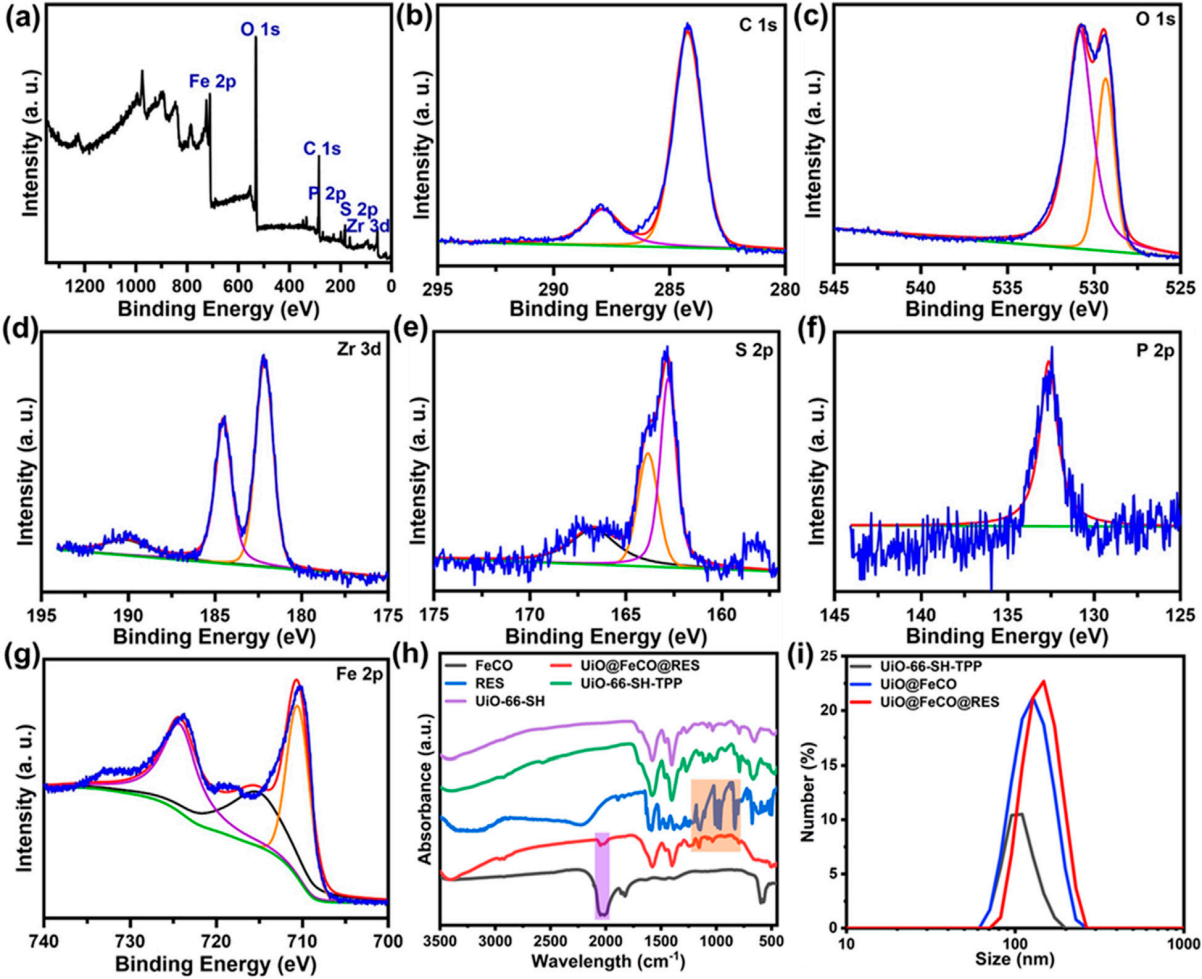
**(A)** XPS spectrum of the UiO@FeCO@RES. High-resolution XPS spectra of **(B)** C 1s, **(C)** O 1s, **(D)** Zr 3d, **(E)** S 2p, **(F)** P 2p, and **(G)** Fe 2p. **(H)** FTIR spectra of FeCO, UiO@FeCO@RES, RES, UiO-66-SH-TPP, and UiO-66-SH. **(I)** Size distribution of UiO-66-SH-TPP, UiO@FeCO, and UiO@FeCO@RES.

To further investigate the composition of UiO@FeCO@RES, an X-ray Photoelectron Spectroscopy (XPS) test was conducted. The survey XPS spectrum revealed that the composition of UiO@FeCO@RES includes C, O, P, S, Zr, and Fe elements (Figure 2A), which is consistent with the results obtained from XRD and EDX. The XPS spectra of C 1s, O 1s, Zr 3d, S 2p, P 2p, and Fe 2p are illustrated in Figures 2B–H, respectively. The peak observed in the P 2p region was attributed to triphenylphosphine in TPP. The high-resolution XPS scan of Fe 2p revealed two peaks at approximately 711.0 and 724.0 eV, corresponding to the FeCO encapsulated within MOFs. Figure 2H presents the Fourier Transform Infrared (FT-IR) spectra of the samples, where the characteristic absorption band for C=O at around 2000 cm^−1^ in the UiO@FeCO@RES confirms the successful encapsulation of FeCO within the MOFs (Zhao et al., 2019; Meng et al., 2020; Zhai et al., 2021). Additionally, Figure 2I shows that dynamic light scattering (DLS) measurements indicated that the average particle size of UiO@FeCO and UiO@FeCO@RES was slightly larger than that of the UiO-66-SH-TPP nanoparticles. Collectively, these results provide strong evidence for the successful loading of FeCO and RES into the MOFs.

### 3.2 RES and CO gas controlled release profiles

**FIGURE 3:**
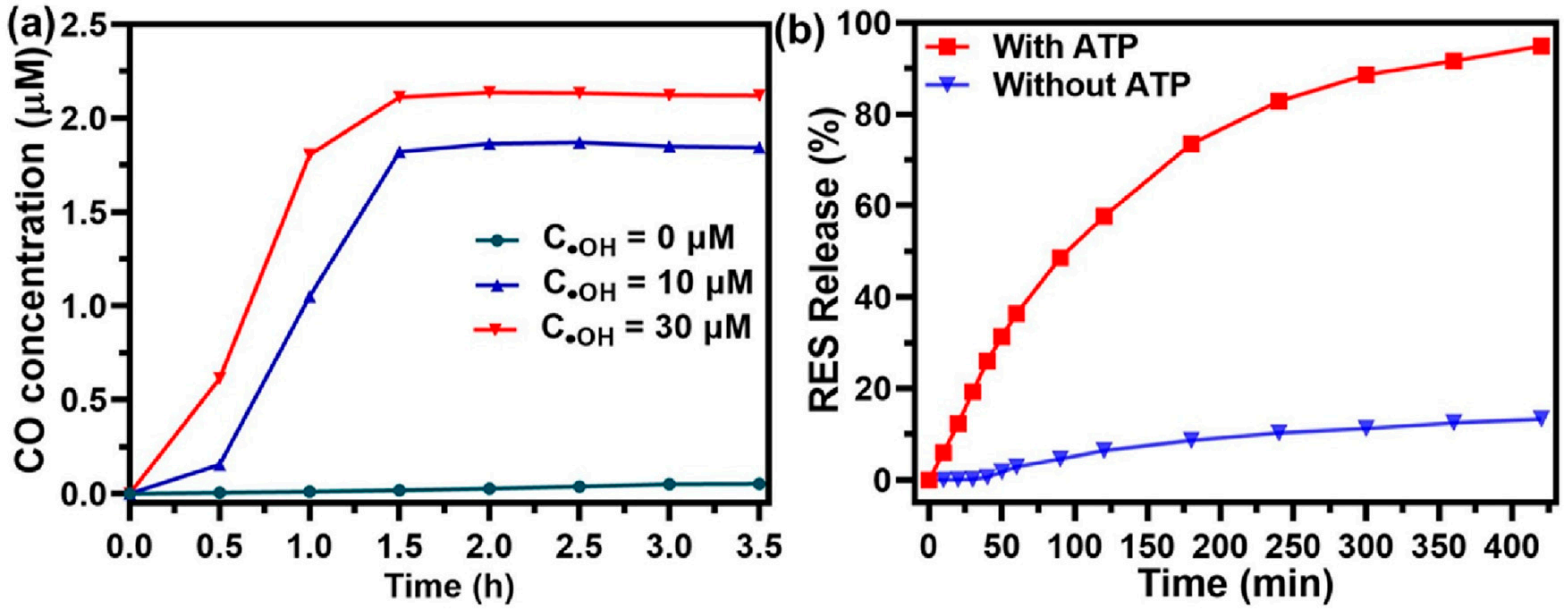
**(A)** Intramitochondrial ROS-responsive CO release profiles of UiO@FeCO@RES at different OH concentrations. **(B)** RES release from UiO@FeCO@RES NPs with or without ATP (1.0 mg/mL).

The behavior of CO release in response to ROS from UiO@FeCO@RES was investigated at various ·OH concentrations, designed to simulate the ROS-rich microenvironment characteristic of mitochondria. The concentration of released CO was monitored using hemoglobin as a probe through the UV-vis spectroscopy (Jin et al., 2018; Wang et al., 2022; Wang et al., 2024). CO gas released from UiO@FeCO@RES under the strong oxidizing effect of ·OH through a Fenton-like reaction, as previously reported. As illustrated in Figure 3A, the release of CO gas was dependent on both the ·OH concentration and time. After 3.5 h, the concentration of released CO gas may reach 1.8 μM at an OH concentration of 10 μM. Furthermore, a higher OH concentration resulted in a more rapid CO release. By calculating UV-vis absorption, the loading of RES in UiO@FeCO@RES nanoparticles was determined to be 3.98%. Duo to the strong metal coordination ability of ATP, UiO@FeCO@RES can disintegrate in an ATP solution (Wan et al., 2020). Consequently, we further explored the drug release behavior of UiO@FeCO@RES in the presence of ATP. As shown in Figure 3B, less than 10% of RES was released in the group without ATP, thereby minimizing the side effects associated with RES leakage. In contrast, over 90% of RES was released within 5 h following ATP addition, indicating ATP-responsive drug “burst release”. These results clearly demonstrate that both CO gas and RES drug can be released from UiO@FeCO@RES within the mitochondrial microenvironment.

### 3.3 Mitochondrial targeting of UiO-66-SH-TPP and CO gas release *in vitro*

**FIGURE 4:**
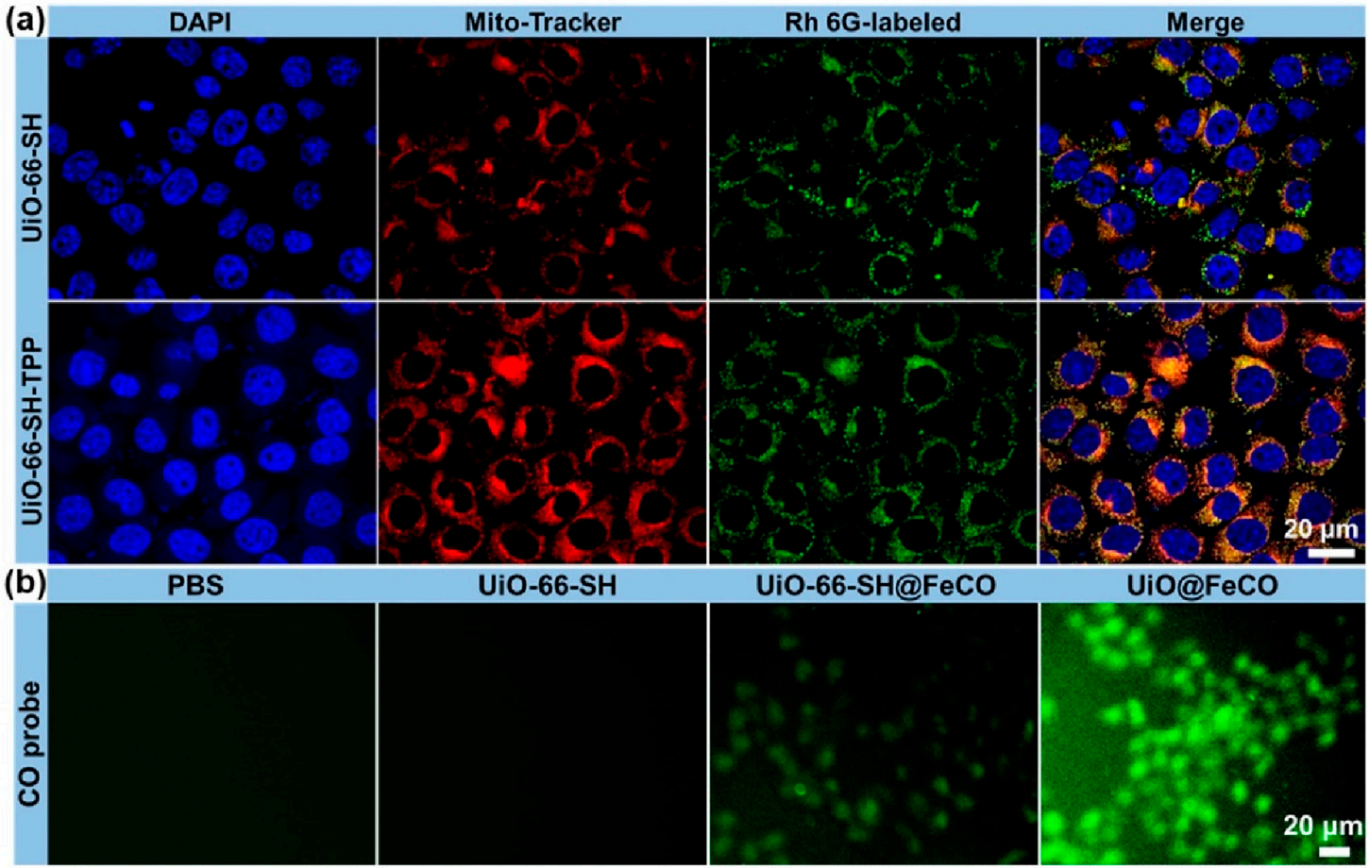
**(A)** Co-localization analysis of HeLa cells and mitochondria. The red fluorescence is from Mito-Tracker, and the green fluorescence is from the Rh 6G-labeled UiO-66-SH and UiO-66-SH-TPP. **(B)** Fluorescent images of HeLa cells after incubation with UiO-66-SH, UIO-66-SH@FeCO, and UiO@FeCO stained with CO probe.

To verify the mitochondria-targeting capability of UiO-66-SH-TPP, colocalization experiments were performed in HeLa cells, with UiO-66-SH serving as the control. As illustrated in Figure 4A, mitochondria are depicted in red, while nuclei are shown in blue. The green fluorescence represents the Rh 6G-labeled UiO-66-SH or UiO-66-SH-TPP. In the merged images, cells treated with UiO-66-SH-TPP exhibited a more pronounced yellow fluorescence overlap compared to the UiO-66-SH group, and the Pearson’s coefficient and overlap coefficient of UiO-66-SH-TPP were 0.86 and 0.88, much higher than those of the UiO-66-SH treated group (Supplementary Figure S2). This result indicated that TPP ligands effectively promote the accumulation of UiO-66-SH-TPP in mitochondria. Additionally, intracellular CO generation was visually assessed using fluorescent imaging with a CO fluorescent probe, following the method proposed by Feng et al. (2017). As demonstrated in Figure 4B, weak but discernible green fluorescence, indicative of CO gas, was observed in HeLa cells incubated with UiO-66-SH@FeCO. In contrast, the green fluorescence significantly increased in cells treated with UiO@FeCO, which could be attributed to the mitochondrial-targeting effects and ROS-responsive behavior of FeCO. Collectively, these results accordantly confirm that the modification of the mitochondria-targeting molecule of TPP facilitates the release of a substantial amounts of CO gas by UiO@FeCO within the mitochondria, thereby enhancing its anti-tumor therapeutic efficacy.

### 3.4 CO gas mediated mitochondrial dysfunction

**FIGURE 5:**
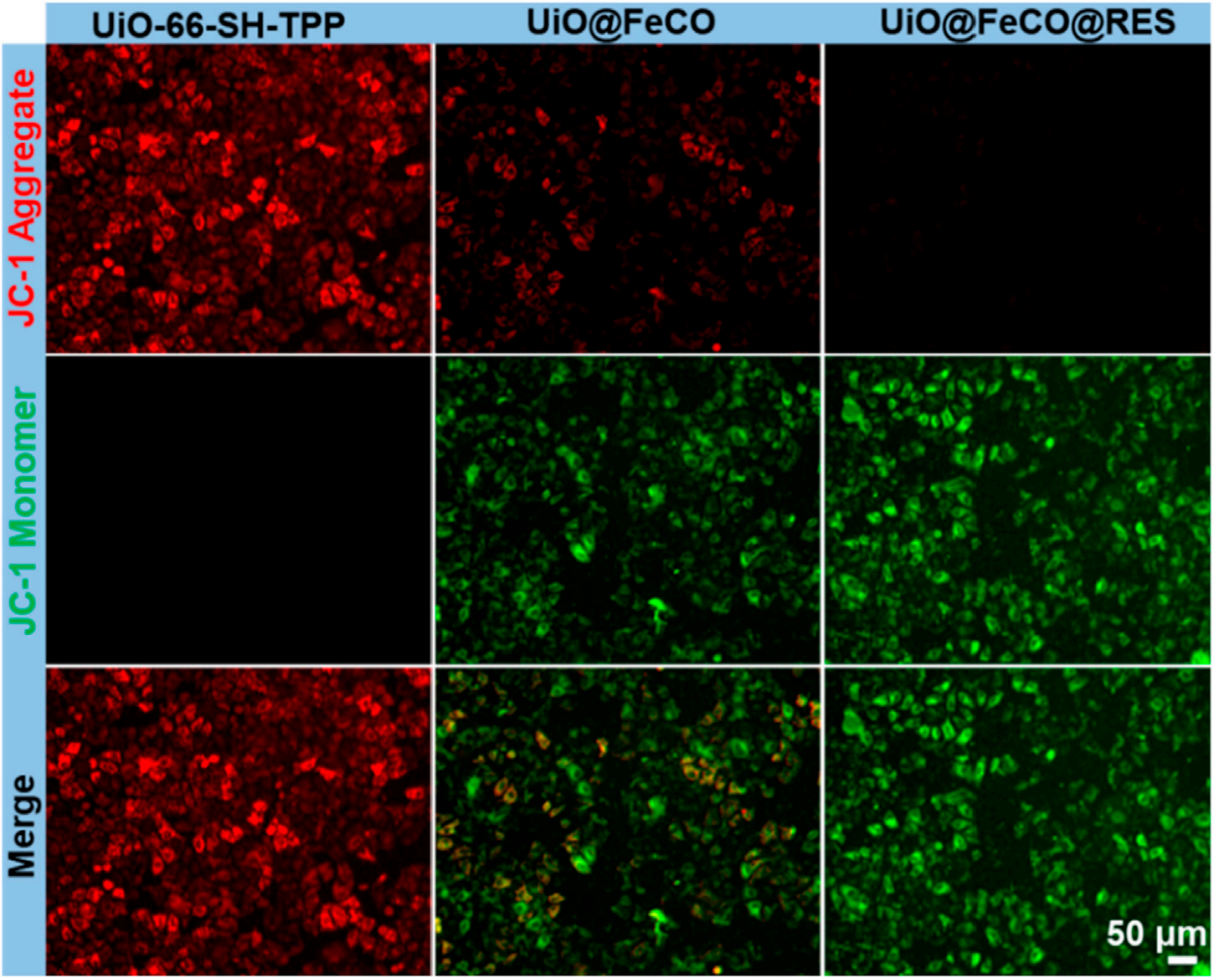
Intracellular MMP detection of HeLa cells by JC-1 after various treatments.

It is well established that the generation of intracellular CO gas can directly lead to mitochondrial dysfunction, which is further exacerbated by RES-mediated suppression of the aerobic respiratory chain (Meng et al., 2020; Wan et al., 2020). To demonstrate this, the mitochondrial membrane potential (MMP) was measured using the JC-1 assay (Liu et al., 2021; Wang et al., 2022). Normal mitochondria exhibit a high MMP, which causes JC-1 to aggregate within mitochondrial matrix, resulting in red fluorescence. Conversely, a decrease in MMP causes a shift in JC-1 fluorescence from red to green, indicating mitochondrial dysfunction. As depicted in Figure 5, HeLa cells treated with UiO-66-SH-TPP displayed extensive red fluorescence. In contrast, the fluorescence of JC-1 changed from red to green in cells treated with UiO@FeCO, likely due to mitochondrial damage caused by the released CO gas in mitochondria. Furthermore, the MMP was further diminished when the cells were co-cultured with UiO@FeCO@RES, suggesting that the release of RES in mitochondria can trigger an ATP-blocked metabolic effect. These results demonstrate that the synergistic effect of RES and CO gas production in UiO@FeCO@RES therapeutic agent may effectively induce mitochondrial damage and subsequent cell death.

### 3.5 *In vitro* CO gas therapy efficacies

**FIGURE 6:**
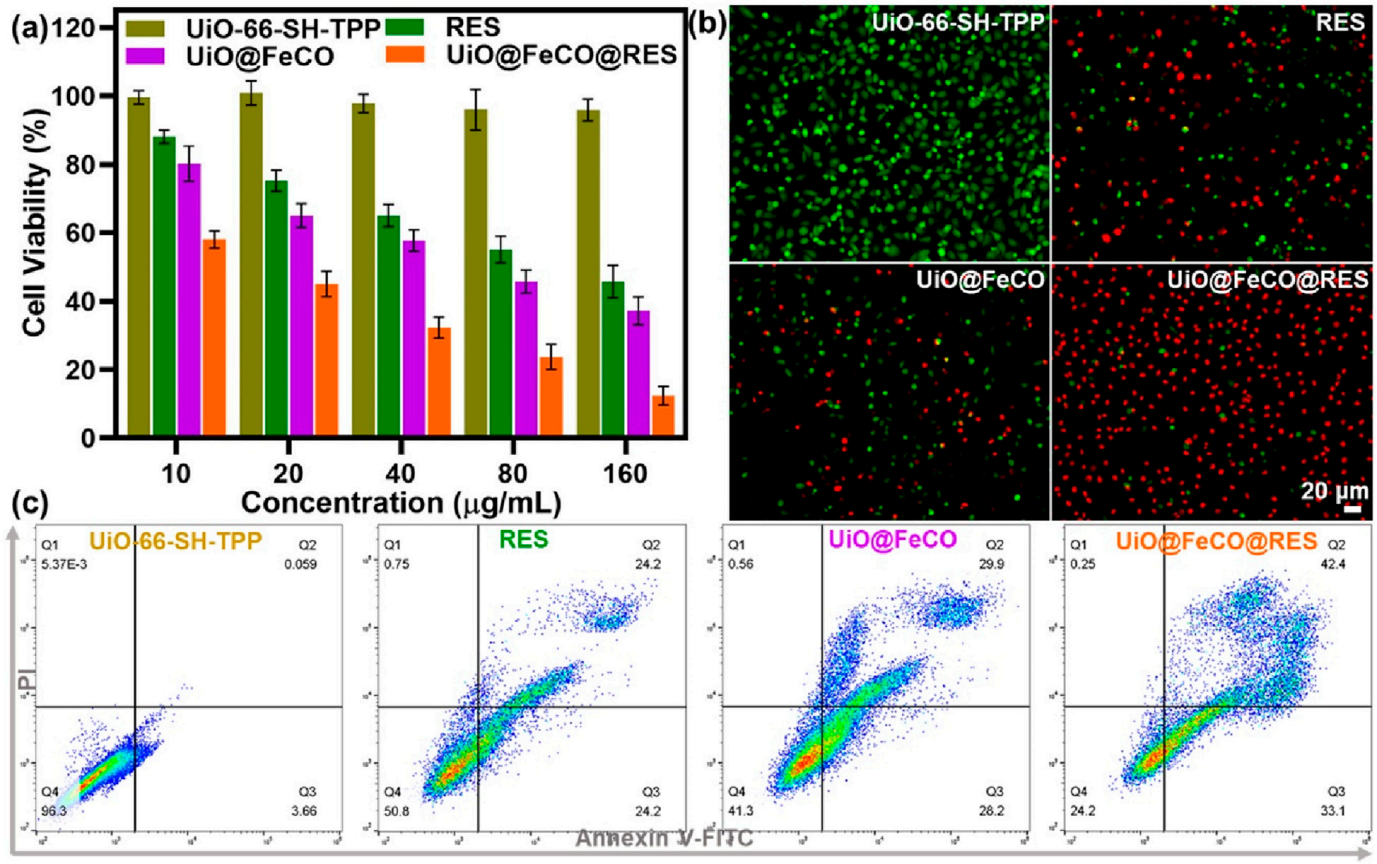
**(A)** Cell viability of HeLa cells under different treatments. **(B)** Live/dead cell staining experiment to visualize the viability of HeLa cells. **(C)** Flow cytometry detection of HeLa cells administered different treatments.

The study examined the anticancer effects of UiO@FeCo@RES on HeLa cells *in vitro* using the standard MTT assay. Figure 6A demonstrates that more than 95% of HeLa cells remained viable after co-incubation with UiO-66-SH-TPP, suggesting that UiO-66-SH-TPP has low toxicity and high biocompatibility. Following the encapsulation of FeCO, the UiO@FeCO complex exhibited considerable anticancer activity. At a concentration of 160 μg/mL, the survival rates of cells treated with UiO@FeCO plummeted to 37%, which is attributed to the anticancer properties of CO gas. Additionally, RES, as a respiratory inhibitor, exhibited its own anti-tumor effects. When integrated into the nanocarriers, the UiO@FeCO@RES group demonstrated significant tumor suppression, as cell viability fell sharply to 12%, underscoring the synergistic anticancer impact of combining RES and CO-gas therapy. Furthermore, we utilized calcein-AM/PI staining to verify the therapeutic impact of UiO@FeCO@RES at the cellular level (Figure 6B). In comparison to the RES and UiO@FeCO groups, HeLa cells treated with UiO@FeCO@RES exhibited the most intense red fluorescence density, indicative of widespread cell death. Moreover, flow cytometry further confirmed the strong combined anti-tumor efficacy of UiO@FeCO@RES (Figure 6C). These results evidenced that the designed nanotherapeutic agent of UiO@FeCO@RES showed excellent biocompatibility and could be used as a potential candidate for anti-tumor therapy.

## 4 Conclusion

In summary, we have successfully designed a ROS-responsive CO gas nanogenerator based on RES and FeCO incorporated MOFs nano-drug delivery system for mitochondria-targeted anticancer therapy. *In vitro* experimental results demonstrated that UiO@FeCO@RES effectively localizes to mitochondria, where it is subsequently disassembled by ATP overexpressed in mitochondria to release RES drug for ATP-blocked metabolic therapy. Concurrently, the intramitochondrial ROS could trigger the CO gas release from UiO@FeCO@RES, facilitating CO therapy. Thus, the prospective therapeutic nano-agent of UiO@FeCO@RES can realize synergistic cancer therapy by integrating RES drug therapy with CO gas therapy. Overall, this work presents an innovative approach that combines gas therapy with RES-mediated suppression of ATPase activity, overcoming the drawbacks of single therapy and offering a novel strategy for CO gas therapy.

## Data Availability

The raw data supporting the conclusions of this article will be made available by the authors, without undue reservation.
