# Supplementary material for: Resveratrol-loaded metal-organic framework for mitochondria-targeted amplified CO gas therapy
Source: Front Chem. 2025 Jan 20;13:1545850. doi: 10.3389/fchem.2025.1545850 (PMC11788274; doi:10.3389/fchem.2025.1545850)
Supplement: Supplementary file 1 [file DataSheet1.docx]

**Supplementary material**

Resveratrol-loaded metal-organic framework for mitochondria-targeted amplified CO gas therapy

Fengqin Wang, Yingfang Jiang, Yang Wang^*^

Nantong Institute of Technology, School of Mechanical Engineering, Nantong, Jiangsu 226006, China.

***Correspondence:**Yang Wang, ywang1985@163.com

1. **Figures**


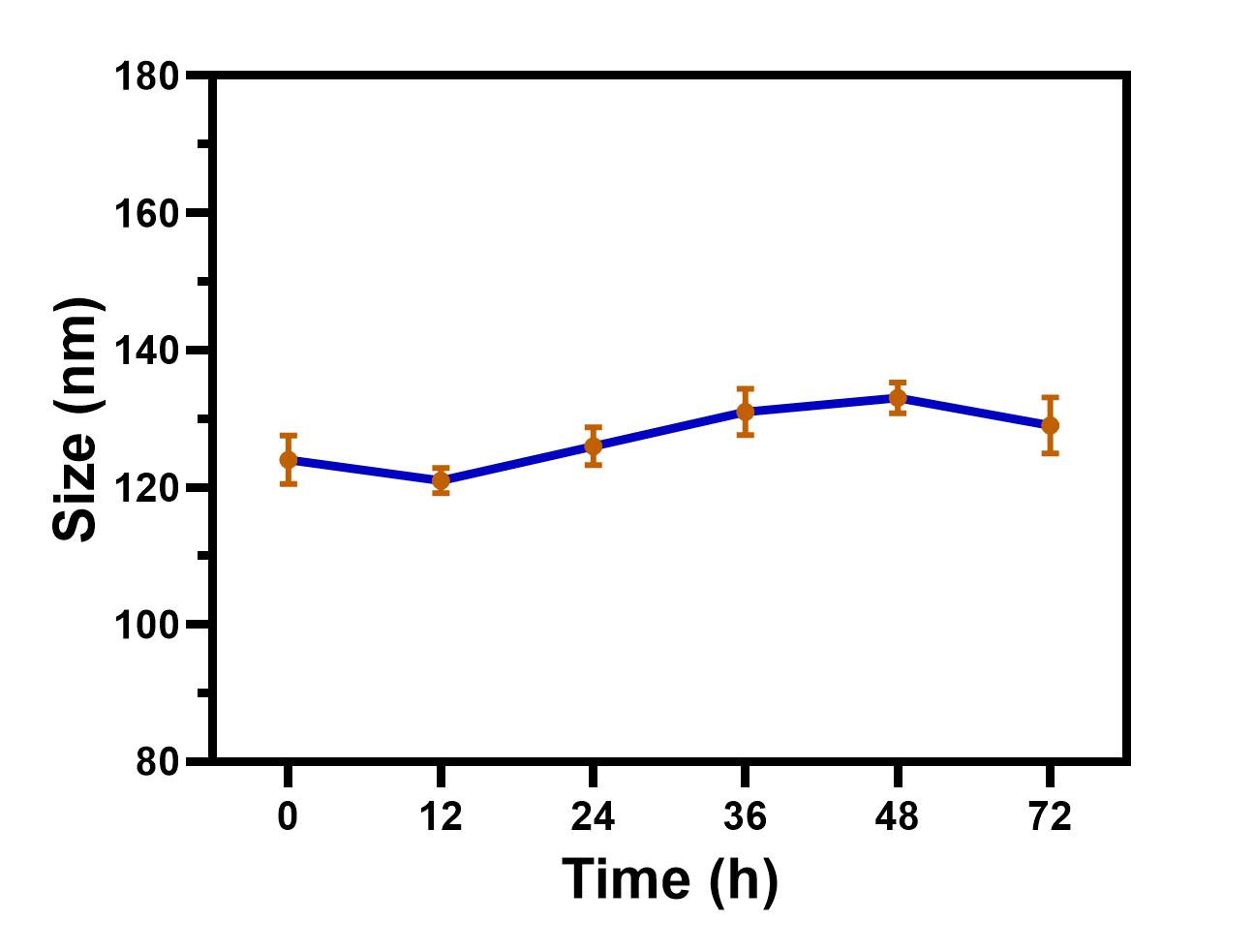


**Figure S1.** The mean particle size changes of UiO@FeCO@RES within 3 days in PBS solution (pH=7.4).


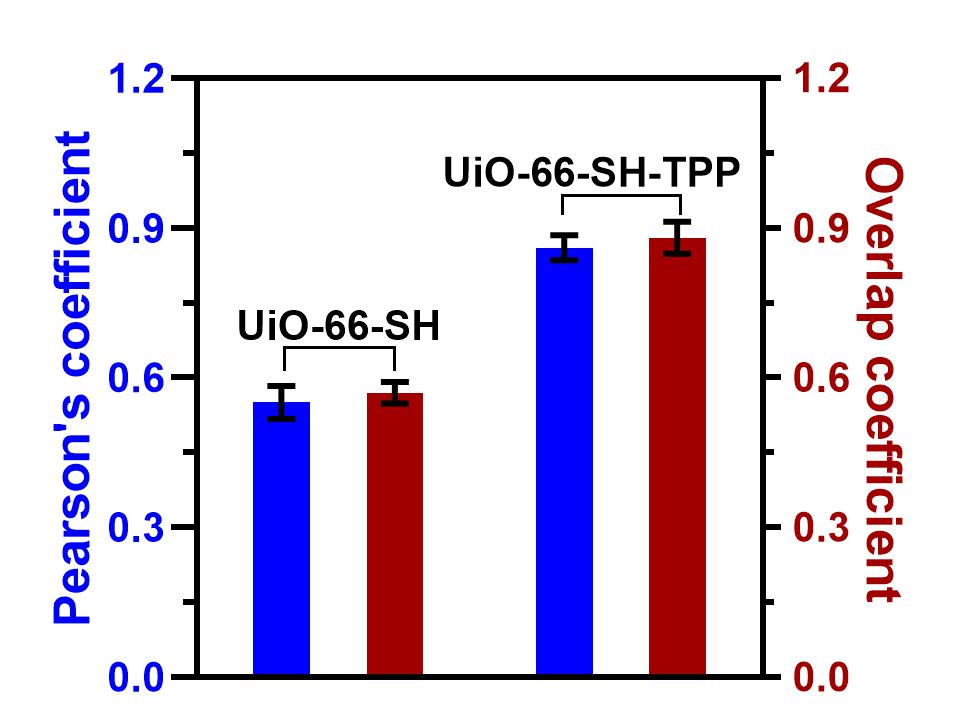


**Figure S2.** The Pearson’s coefficient and overlap coefficient between the UiO-66-SH or UiO-66-SH-TPP green fluorescence and Mito-Tracker Red fluorescence, based on the typical images in Figure 4a.
